# Total Sesquiterpene Glycosides from Loquat Leaves Ameliorate HFD-Induced Insulin Resistance by Modulating IRS-1/GLUT4, TRPV1, and SIRT6/Nrf2 Signaling Pathways

**DOI:** 10.1155/2021/4706410

**Published:** 2021-10-27

**Authors:** Ruoyun Wu, Tunyu Jian, Xiaoqin Ding, Han Lv, Xiuhua Meng, Bingru Ren, Jing Li, Jian Chen, Weilin Li

**Affiliations:** ^1^Institute of Botany, Jiangsu Province and Chinese Academy of Sciences, Nanjing 210014, China; ^2^Department of Food Science and Technology, College of Light Industry and Food Engineering, Nanjing Forestry University, Nanjing 210037, China; ^3^Co-Innovation Center for Sustainable Forestry in Southern China, Forestry College, Nanjing Forestry University, Nanjing 210037, China

## Abstract

Loquat (*Eriobotrya japonica* Lindl.), a subtropical fruit tree native to Asia, is not only known to be nutritive but also beneficial for the treatment of diabetes in the south of China. To expand its development, this study was undertaken concerning the potential therapeutic role of total sesquiterpene glycosides (TSGs) from loquat leaves in insulin resistance (IR), the major causative factor of type 2 diabetes mellitus (T2DM). Male C57BL/6 mice were fed on high-fat diet (HFD) to induce IR and then were given TSG by oral administration at 25 and 100 mg/kg/day, respectively. TSG notably improved metabolic parameters including body weight, serum glucose, and insulin levels and prevented hepatic injury. Moreover, inflammatory response and oxidative stress were found to be remarkably alleviated in IR mice with TSG supplement. Further research in liver of IR mice demonstrated that TSG repaired the signalings of insulin receptor substrate-1 (IRS-1)/glucose transporter member 4 (GLUT4) and AMP-activated protein kinase (AMPK), which improved glucose and lipid metabolism and prevented lipid accumulation in liver. It was also observed that TSG suppressed the expression of transient receptor potential vanilloid 1 (TRPV1), whereas the signaling pathway of sirtuin-6 (SIRT6)/nuclear factor erythroid 2-related factor 2 (Nrf2) was significantly promoted. Based on the results, the current study demonstrated that TSG from loquat leaves potentially ameliorated IR *in vivo* by enhancing IRS-1/GLUT4 signaling and AMPK activation and modulating TRPV1 and SIRT6/Nrf2 signaling pathways.

## 1. Introduction

Owing to the popularity of fast food and takeout, dietary habits in the form of high fat are so common that metabolic disorders are prevalently increasing worldwide. Insulin resistance (IR) is regarded as a main pathological feature of metabolic disorders, such as obesity, type 2 diabetes mellitus (T2DM), and nonalcoholic fatty liver disease (NAFLD). IR is a complex pathological condition, which refers to the inadequate response of insulin target tissues during insulin stimulation. The molecular mechanism of IR is not fully understood, but it is confirmed that imbalanced glucose and lipid metabolism, inflammation, and oxidative stress are major factors associated with the development of IR [1–3].

In normal state, insulin binds to the insulin receptor, and then, insulin receptor substrate-1 (IRS-1) is phosphorylated. It activates its downstream targets including glucose transporter member 4 (GLUT4) and regulates blood glucose balance [4]. AMP-activated protein kinase (AMPK) is a vital regulator in the metabolism of glucose and lipid [5]. AMPK has been believed to be a therapeutic target for many metabolic disorders including IR [6, 7]. Moreover, a few earlier studies have revealed that the activated AMPK could positively regulate the expression of GLUT4 [8, 9]. The suppression of AMPK and IRS-1/GLUT4 has been proven to be associated with dysregulated glucose and lipid metabolism in IR-related models [9–11]. Conversely, molecules that could upregulate IRS-1/GLUT4 signaling or activate AMPK always display great effects on IR [10, 12, 13].

Transient receptor potential vanilloid 1 (TRPV1) is a nonselective cation channel, which is associated with inflammation, nociception, and thermosensation [14]. TRPV1 can be activated by heat, pH, and chemicals. As a member of chemicals, natural products were also found to interact with TRPV1, including vanilloids, flavonoids, terpenoids, and cannabinoids [15]. TRPV1 is not only localized to sensory neurons but also present in nonneuronal tissues. Hence, it plays pivotal roles in many disease progressions, such as inflammation in chronic obstructive pulmonary disease, oxidant stress-induced pain, and neuronal injury [16–18]. Recently, TRPV1 has drawn rising attention with its role in metabolic disorders [19–21]. TRPV1 deletion displayed anti-inflammatory effect in murine models involving metabolism disorder [22]. Accordingly, targeting TRPV1 may be a useful therapy for IR.

Sirtuin-6 (SIRT6) is a nuclear protein expressed in all of tissues, including liver, heart, and adipose [23–25]. Since its discovery, SIRT6 has been substantiated that it positively involved in numerous physiological processes. Furthermore, it has been regarded as a therapeutic agent for IR [26, 27]. SIRT6 deficiency could make diet-induced IR heavier [28]. SIRT6 has been well established to promote nuclear factor erythroid 2-related factor 2 (Nrf2) binding the antioxidant response elements (AREs) and to protect cells from oxidative stress [29]. Additionally, robust expression of SIRT6 has been shown an inhibitory effect on TRPV1-regulated inflammation [30, 31]. Thus, promoting the SIRT6/Nrf2 signaling will be a distinctive strategy to curb IR.

Loquat (*Eriobotrya japonica* Lindl.) is a subtropical fruit tree that is widely distributed in China. Its leaf is a new source of food approved by the Ministry of Health of the People's Republic of China in 2014. Besides, loquat leaf has been found to possess beneficial properties such as anti-inflammatory, antioxidant effects, and improvement of metabolic diseases [16, 32]. Mounting evidence has shown that different extracts from loquat leaves were helpful in controlling IR. The triterpenoid acids from loquat leaves have been demonstrated to act on signalings of AMP-activated protein kinase (AMPK), insulin receptor substrate-1 (IRS-1), and Nrf2, which corrected abnormity of glucose and lipid metabolism in high-fat diet (HFD) mice and contributed to alleviating IR [33, 34]. As previously reported, the total sesquiterpene glycosides isolated from loquat leaves were considered to be the possible secondary metabolites responsible for its antidiabetic activity [35]. However, it is elusive that how these total sesquiterpene glycosides (TSGs) affect the glucose and lipid metabolism, inflammation, and oxidative stress *in vivo* under IR condition.

To expand their development, this study was undertaken concerning the potential therapeutic role of TSG from loquat leaves in IR, the major causative factor of T2DM. The present study confirmed the effects of TSG on HFD-induced IR *in vivo* and explored its underlying mechanism.

## 2. Materials and Methods

### 2.1. Preparation and Analysis of TSG from Loquat Leaves

Total sesquiterpene glycosides from loquat leaves were prepared in our lab following the method that we reported earlier [36, 37]. In brief, the powder of dried loquat leaves was percolated with 80% ethanol solution twice. The combined extracts were concentrated and centrifuged. Next, the supernatant was evaporated and subjected to column chromatography on macroporous resin XAD16, using solvent gradient system from H_2_O to EtOH. The 60% and 70% EtOH eluted fractions were further column chromatographed over polyamide eluting with solvent gradient system from H_2_O to MeOH. The H_2_O eluted fraction was further column chromatographed by RP-C18 with different mixtures of H_2_O and MeOH to obtain TSG.

**Figure 1:**
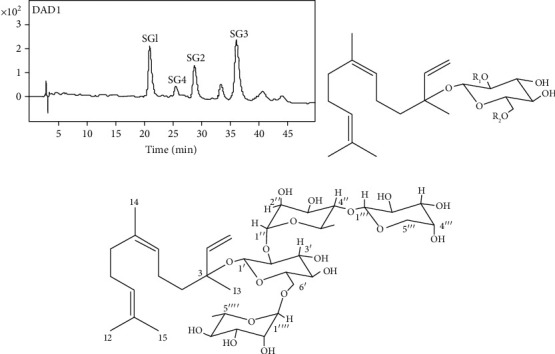
Compositional analysis of TSG from loquat leaves. (a) Chromatograms of the constituents through HPLC. (b) Chemical structure of SG1-SG3, SG1: *R*_1_ = Rha (1⟶4) Rha, *R*_2_ = Rha; SG2: *R*_1_ = Rha (1⟶4) Rha, *R*_2_ = H; and SG3: *R*_1_ = Rha, *R*_2_ = Rha. (c) Chemical structure of SG4.

HPLC was performed on Dionex Ultimate 3000 HPLC systems (Thermo Fisher Scientific Inc., Germany), equipped with a quaternary solvent delivery pump, an autosampler, DAD detector, and a Chromeleon Workstation. The analytical column Inertsil ODS-SP (4.6 mm × 250 mm, 5 *μ*m) was used, and the temperature of column was kept at 35°C. The mobile phase was composed of methanol (A) and 0.1% formic acid-water (B) with gradient elution (0-10 min, 57% A; 12-20 min, 58% A; 20-35 min, 60% A; and 35-50 min, 65% A). The flow rate was 0.5 mL/min, and the elute was monitored at 210 nm. For quantification, nerolidol-3-*O*-*α*-l-rhamnopyranosyl-(1→4)-*α*-l-rhamnopyranosyl-(1→2)-[*α*-l-rhamnopyranosyl-(1→6)]-*β*-D-glucopyranoside (SG1), nerolidol-3-*O*-*α*-l-rhamnopyranosyl-(1→4)-*α*-l-rhamnopyranosyl-(1→2)-*β*-D-glucopyranoside (SG2), nerolidol-3-*O*-*α*-l-rhamnopyranosyl-(1→2)-[*α*-l-rhamnopyranosyl-(1→6)]-*β*-D-glucopyranoside (SG3), and nerolidol-3-O-*α*-l-arabinopyranosyl-(1→4)-*α*-l-rhamnopyranosyl-(1→2)-[*α*-l-rhamnopyranosyl-(1→6)]-*β*-D-glucopyranoside (SG4) were purified as standard compounds, and the same conditions were applied to HPLC. The chemical structure of SG1-4 is shown in Figures 1(b) and 1(c) and was consistent with the reported literatures [35, 37–39].

### 2.2. Reagents and Antibodies

The assay kits of glucose, triglycerides (TGs), total cholesterol (TC), low-density lipoprotein cholesterol (LDL-C), high-density lipoprotein cholesterol (HDL-C), superoxide dismutase (SOD), malondialdehyde (MDA), aspartate aminotransferase (AST), alanine aminotransferase (ALT), and Oil Red O staining were obtained from Nanjing Jiancheng Bioengineering Institute, China. Enzyme-linked immuno-sorbent assay (ELISA) kits of tumor necrosis factor-*α* (TNF-*α*), interleukin (IL)-1*β*, and IL-6 were obtained from Lianke Biotechnology, Hangzhou, China. ELISA kit of insulin was purchased from Elabscience Biotechnology, Wuhan, China. The kit for HE staining was purchased from Solarbio, Beijing, China. The BCA assay kit was obtained from Biosharp, China.

Primary antibodies for TRPV1 (1 : 1000) and SOD1 (1 : 200) were from Santa Cruz Biotechnology, USA. Antibodies for SIRT6 (1 : 1000), glucose transporter type 4 (GLUT4, 1 : 1000), SOD2 (1 : 2000), and GAPDH (1 : 20000) were obtained from ProteinTech, China. Antibodies for Nrf2 (1 : 1000), phosphor-IRS-1 (Tyr895) (1 : 1000), and IRS-1 (1 : 1000) were purchased from Cell Signaling Technology, USA. Anti-AMPK (1 : 1000) and anti-phosphor-AMPK (1 : 1000) were purchased from Bioworld Technology, China. Second antibodies for rabbit-source and mouse-source were obtained from Abmart, Shanghai, China.

### 2.3. Animals and Treatment

Six-week-old healthy male C57BL/6 mice weighting 17-20 g were obtained from Shanghai Sino-British SIPPR/BK Lab Animal Co., Ltd., China. All the mice studied were maintained on a 12 h light/dark cycle at 23 ± 2°C with free access to water and food throughout the experiment. Mice were separated into four random groups with eight mice in each group as follows: regular diet (CON), HFD (HFD), low-dose TSG (25 mg/kg) with HFD (HFD+TSG-L), and high-dose TSG (100 mg/kg) with HFD (HFD+TSG-H). After a week of acclimation, the CON group was treated with regular diet for 8 weeks, which contained 20% (weight/weight) flour, 10% rice flour, 20% corn, 26% drum head, 20% bean, 2% fish powder, and 2% bone powder (XieTong Organism Inc., China). Simultaneously, to induce a mouse model with basic pathophysiology of IR, the other groups were provided with HFD for 8 weeks, which were made in our lab (18% lard (w/w), 5% egg powder, 1% cholesterol, 20% sucrose, 0.1% bile salt, and 55.9% regular diet). During the last 4 weeks of induction, groups of HFD+TSG-L and HFD+TSG-H were administered with oral gavages of 25 and 100 mg/kg TSG, respectively. Correspondingly, the groups of CON and HFD received equal volumes of saline by intragastric administration during the last 4 weeks. Mouse body weights were monitored weekly. All the experimental procedures and animal treatments were performed in accordance with the Guide for the Care and Use of Laboratory Animals.

### 2.4. Sample Collection

After 8 weeks of treatment, mice were fasted for 12 h before sacrificed for blood and liver samples. The blood samples were collected by cardiac puncture and centrifuged at 2500 × g for 15 min at 4°C to obtain serum and then stored at -80°C for further experiment. The liver tissues were dissected, photographed, weighed, frozen in liquid nitrogen, and stored at -80°C until analysis.

### 2.5. Biochemical Analysis

The levels in serum of glucose, TG, TC, LDL-C, HDL-C, MDA, SOD, ALT, and AST were measured with commercial kits and analyzed by a Molecular Devices Spectra Max Plus automatic plate reader (Molecular Device, Sunnyvale, CA, USA). The levels of TNF-*α*, IL-1*β*, IL-6, and insulin in serum were tested with ELISA kits following the manufacturer's protocol.

### 2.6. Hematoxylin-Eosin Staining and Oil Red O Staining

For hematoxylin-eosin (HE) staining, liver samples were fixed in formalin buffer, then embedded in paraffin blocks, and sectioned. Sliced sections were stained with hematoxylin and eosin. Pathological changes were observed using a light microscope (Olympus, Japan). For Oil Red O staining, the frozen liver sections were fixed in 10% formaldehyde for 10 min, then washed by isopropanol, stained in Oil Red O, and processed for hematoxylin counter staining. Photomicrographs of tissue sections were taken using a light microscope.

### 2.7. Western Blotting

Liver tissues were first lysed on ice using RIPA lysis buffer and then centrifuged. Protein concentrations were determined using a protein assay kit, with BSA as standards. Equal amount of proteins was electrophoresed on 10% SDS-polyacrylamide gel and transferred onto a 0.45 *μ*m PVDF membrane. Blocked for 2 h with TBST containing skimmed milk powder at room temperature, the membranes were incubated with primary antibodies separately overnight at 4°C. Then, membranes were washed with TBST for 3 times and incubated with secondary antibodies at room temperature for 1 h. Signals were detected by chemiluminescence using the ECL detection reagent. The bands were quantified and analyzed by the Image J software (National Institutes of Health, USA).

### 2.8. Statistics Analysis

Data were presented as mean ± SEM. with GraphPad Prism (GraphPad Software Inc., USA). One-way ANOVA with Tukey's multiple comparison test was used to analyze intergroup significance. *P* < 0.05 was considered to be statistically significant.

## 3. Results

### 3.1. Compositional Analysis of TSG

Four known sesquiterpene glycosides were detected in the TSG sample (Figure 1(a)). The content of sesquiterpene glycosides in TSG was 84.2% ± 2.8%.

### 3.2. TSG Ameliorated Weight Gain and Hepatic Damage Induced by HFD in IR Mice

**Figure 2:**
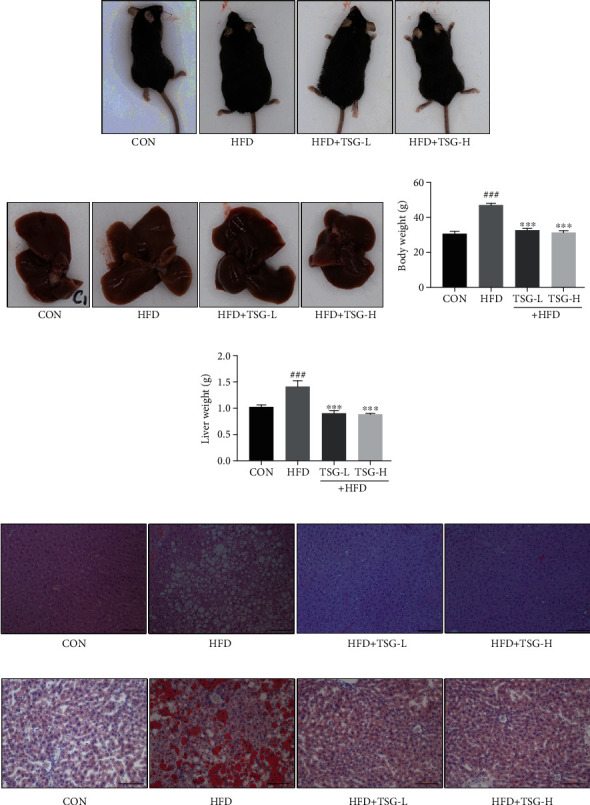
Effects of TSG on body weight, liver weight, and hepatic pathological changes. (a, b) Pictures of mice's bodies and liver tissues. (c, d) Weights of body and liver. (e, f) Liver tissues stained with HE and Oil O Red. The size bar in photomicrograph was 100 *μ*m. Data were presented as mean ± SEM. ^###^*P* < 0.001 vs. mice in the CON group. ^∗∗∗^*P* < 0.001 vs. mice in the HFD group.

**Figure 3:**
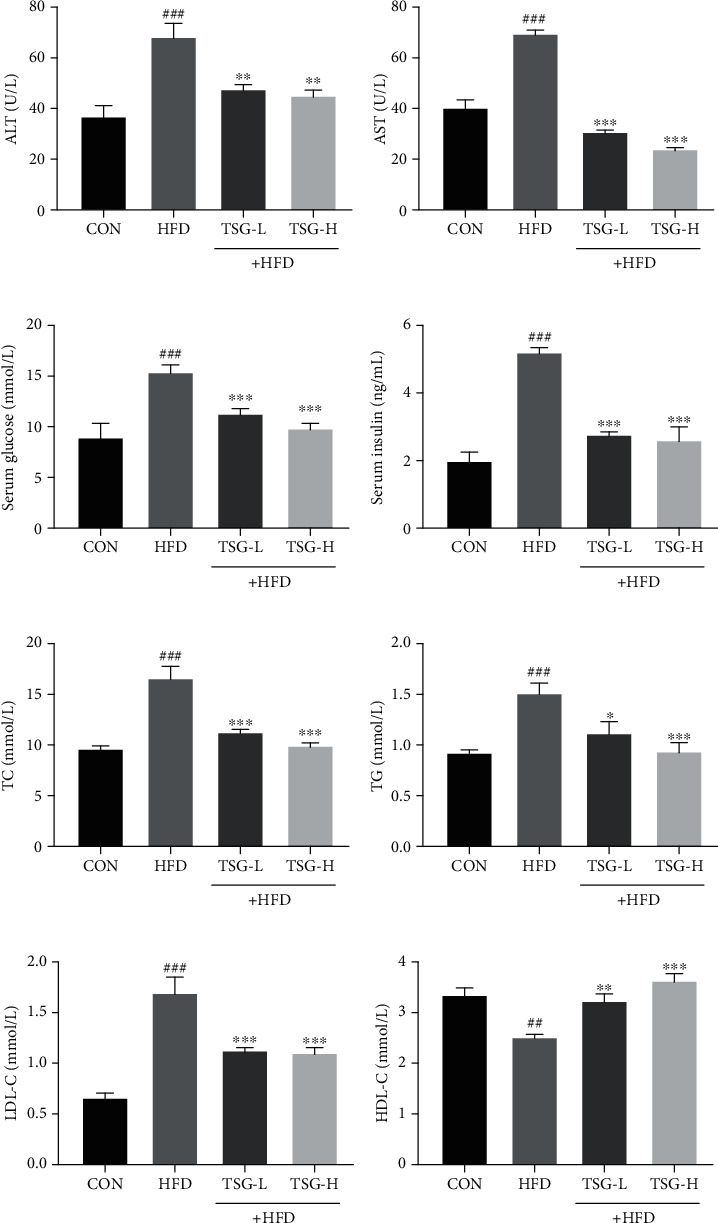
Effects of TSG on metabolic parameters and aminotransferase levels. Activities of (a) ALT and (b) AST. Quantitation of (c) glucose and (b) insulin in serum. Quantitation of (e) TC, (f) TG, (g) LDL-C, and (h) HDL-C. All data were presented as mean ± SEM. ^##^*P* < 0.01 and ^###^*P* < 0.001 vs. mice in the CON group. ^∗^*P* < 0.05, ^∗∗^*P* < 0.01, and ^∗∗∗^*P* < 0.001 vs. mice in the HFD group.

After diet intervention, mice in HFD group were evidently fatter than mice in the CON group (Figures 2(a) and 2(c)). The liver tissues were dark brown both in CON and TSG groups while those were yellowish and significantly heavier in HFD mice (Figures 2(b) and 2(d)). Oral administration of TSG efficaciously reduced body and liver weights of HFD mice, despite low or high doses (*P* < 0.001, Figures 2(c) and 2(d)). Liver is an insulin-sensitive organ with a vital role in maintaining metabolism. When IR develops, abnormal lipid accumulation usually occurs in liver. Hence, HE and Oil Red O staining were performed to evaluate the hepatic pathology and morphology, assessing the effect of TSG on mouse liver. There were dramatical increase of vacuoles and lipid droplets in liver of the HFD group. TSG administration enormously lessened vacuoles and lipid droplets in liver tissues (Figures 2(e) and 2(f)). As important supplements to the histological changes of liver, the activities of ALT and AST were tested to monitor liver function. Activities of ALT and AST were dramatically elevated in the HFD mice, which were significantly decreased after TSG treatment (*P* < 0.01, Figures 3(a) and 3(b)).

### 3.3. TSG Relieved Hyperglycemia and Hyperlipidemia in IR Mice

Hyperglycemia and hyperlipidemia are common in IR patients. Compared with the mice on regular diet, excessive serum insulin and glucose were detected in mice on HFD. The HFD-driven increases of serum insulin and glucose were both reduced in mice treated with TSG at 25 and 100 mg/kg (*P* < 0.001, Figures 3(c) and 3(d)). Next, we assayed the concentration of TC, TG, LDL-C, and HDL-C in serum to confirm the hypolipidemic function of TSG in IR mice. The levels of TC and TG were highly stimulated by HFD, and similar trend was found in serum LDL-C level (*P* < 0.001, Figures 3(e)–3(g)). In the meantime, lower serum HDL-C level was detected in the HFD group (*P* < 0.01, Figure 3(h)). We found that TSG treatment achieved hypolipidemic effect, as indicated by the reduction of TC, TG, and LDL-C and elevation of HDL-C in serum.

### 3.4. Anti-Inflammatory and Antioxidant Effects of TSG in IR Mice

**Figure 4:**
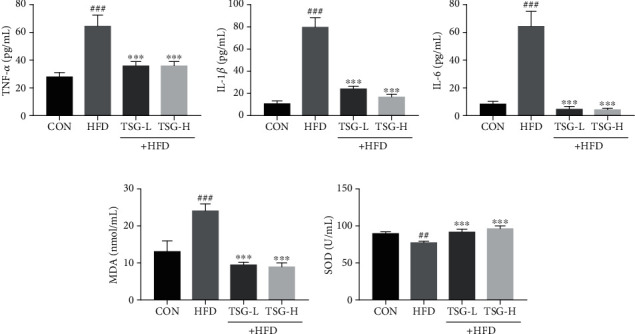
Anti-inflammatory and antioxidant effects of TSG. Quantitation of (a) TNF-*α*, (b) IL-1*β*, and (c) IL-6 in serum. (d) The content of MDA in serum. (e) Activity of SOD in serum. All data were presented as mean ± SEM. ^##^*P* < 0.01 and ^###^*P* < 0.001 vs. mice in the CON group. ^∗∗∗^*P* < 0.001 vs. mice in the HFD group.

Systemic inflammation in mice was estimated by the content of inflammatory cytokines using ELISA kits. Compared with regular-diet mice, each concentration of TNF-*α*, IL-1*β*, and IL-6 in serum was dramatically higher in HFD mice. These inflammatory cytokines were enormously reduced in TSG-treatment groups (Figures 4(a)–4(c)).

MDA, a landmark product of lipid peroxidation, was overproduced in mice by continuous HFD treatment. In addition, HFD consumption suppressed the activity of SOD which was identified as one of antioxidant defense system. Supplementation of TSG induced significant reduction in serum MDA content while remarkable increase in SOD activity (*P* < 0.001, Figures 4(d) and 4(e)).

### 3.5. Effect of TSG on Hepatic IRS-1/GLUT4 and AMPK Signaling Pathways

**Figure 5:**
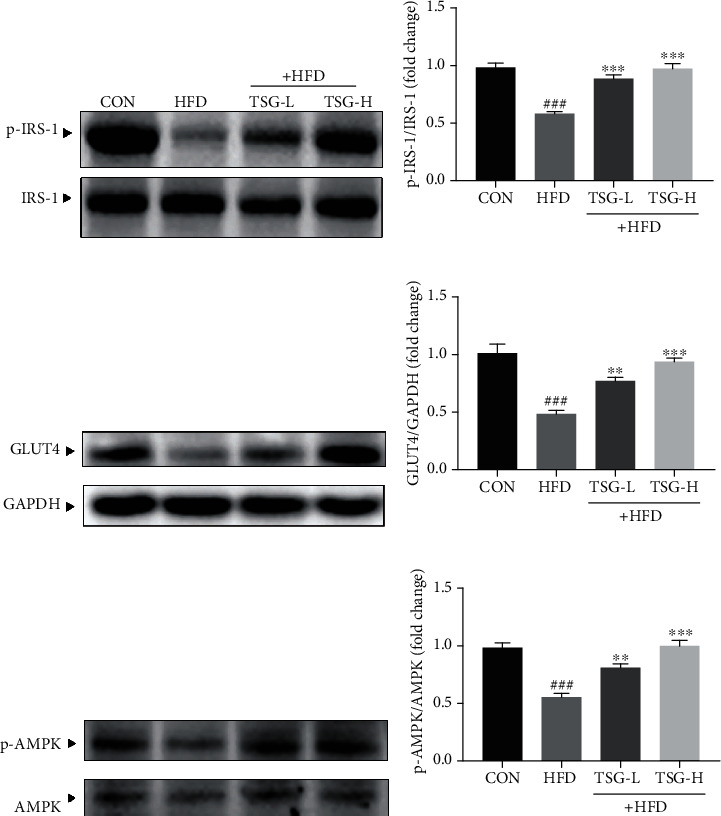
Enhanced signaling of IRS-1/GLUT4 and AMPK following TSG administration. (a, b) The phosphorylated and total forms of IRS-1 in liver. (c, d) GLUT4 expression in liver tissue. (e, f) The phosphorylated and total forms of AMPK in liver. All data were presented as mean ± SEM. ^###^*P* < 0.001 vs. mice in the CON group. ^∗∗^*P* < 0.01 and ^∗∗∗^*P* < 0.001 vs. mice in the HFD group.

HFD-induced IR markedly impaired insulin signaling, including the decrease of IRS-1 tyrosyl phosphorylation and GLUT4 expression. TSG treatment revealed an apparent improvement in p-IRS-1 and GLUT4 expression compared with the HFD group (Figures 5(a)–5(d)). Meanwhile, TSG significantly reversed HFD-induced suppression of AMPK phosphorylation in IR mice (Figures 5(e) and 5(f)).

### 3.6. Effect of TSG on Regulating Hepatic TRPV1

**Figure 6:**
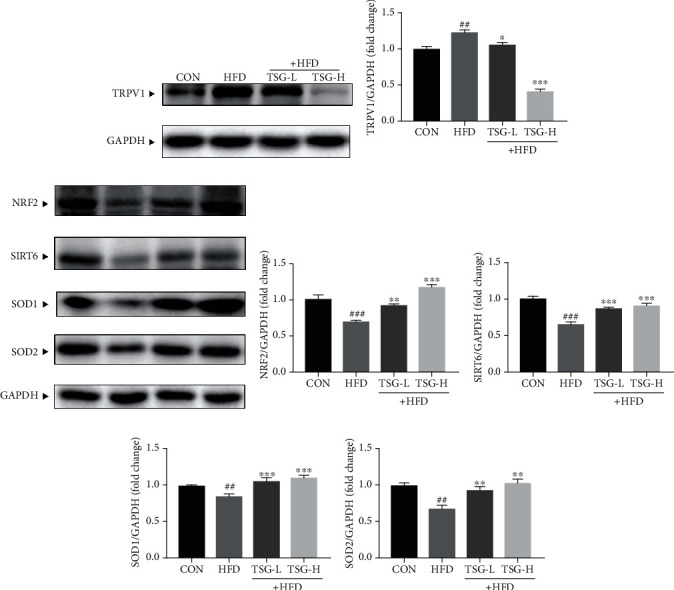
Effects of TSG on the TRPV1 and SIRT6/Nrf2 signaling pathways. (a, b) The protein expression of TRPV1 in livers. (c–g) The protein expression of Nrf2, SIRT6, SOD1, and SOD2 in liver tissues. Data were presented as mean ± SEM. ^##^*P* < 0.01 and ^###^*P* < 0.001 vs. mice in the CON group. ^∗^*P* < 0.05, ^∗∗^*P* < 0.01, and ^∗∗∗^*P* < 0.001 vs. mice in the HFD group.

Based on the role of TRPV1 in inflammation and metabolism, we continued to analyze whether TSG prevented the IR-caused damage through TRPV1 on protein level. TRPV1 expression of the HFD group was greatly raised in comparison with the CON group. Hepatic TRPV1 expression was effectively reduced in mice treated with TSG, especially with the high dose of TSG (Figures 6(a) and 6(b)).

### 3.7. Effect of TSG on SITR6/Nrf2 Signaling

Considering the former reports suggested that SIRT6 promoted Nrf2 signaling in respond to oxidative stress, we also verified whether TSG had a protective function in HFD-induced IR by regulating SIRT6/Nrf2 pathway. Protein expressions of both SIRT6 and Nrf2 were greatly inhibited in livers of HFD mice. In accordance with Nrf2, protein levels of SOD1 and SOD2 were lower than those in the CON group. After TSG treatment, all the decreased trends were distinctly reversed (Figures 6(c)–6(g)).

## 4. Discussion

Loquat (*Eriobotrya japonica* Lindl.) leaf is used as not only a delicious tea but also a kind of diet-therapy food with a rich resource in China. As traditional Chinese medicine, loquat leaf now is clinically used to treat cough and has been recorded in history for hundreds of years. Besides, it has therapeutic potential for T2DM and obesity. The bioactivities of loquat leaf extracts or compounds have been authenticated scientifically and methodically by researchers, including anti-inflammation and antioxidation [16, 40–42]. According to earlier studies, extracts of loquat leaves reduced TC and promoted glucose uptake in HFD-induced IR condition, which was instrumental in treating IR [43, 44]. Our previous investigation indicated that sesquiterpene glycosides from loquat leaves exhibited the bioactivity of reducing lipid accumulation and enhancing glucose uptake in insulin-resistant HepG2 cells via AMPK pathway [37].

**Figure 7:**
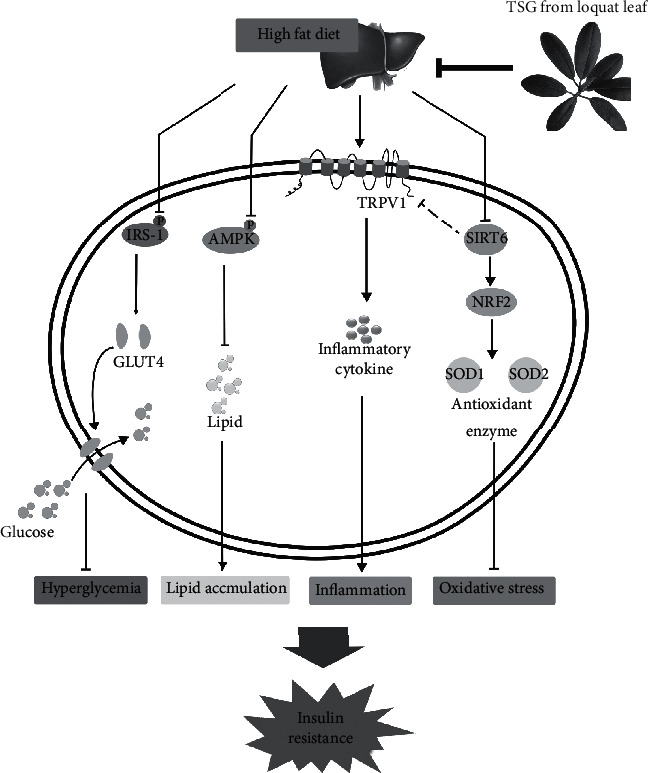
A schematic illustration of major points of conclusion.

Insulin resistance is defined as failure of insulin-target tissues to respond to insulin stimulation. IR induced by HFD disrupts normal regulation mechanism of glucose and lipid, leading to hyperglycemia and abnormal lipid accumulation in nonadipose tissues [45]. In this study, TSG extracted from loquat leaves effectively prevented HFD-induced obesity and irregular glucose and lipid metabolism, showing protective effect against IR. And the protection of TSG was also considered to be associated with anti-inflammation and antioxidation. The modulation of IRS-1/GLUT4, AMPK, TRPV1, and SIRT6/Nrf2 signal pathways might be involved in the potential molecular mechanism (Figure 7).

Liver is a unique organ in maintaining glucose and lipid homeostasis. IR is usually accompanied by lipid accumulation in liver and even steatosis. Our data confirmed that TSG had hypolipidemic effect and prevented abnormal lipid accumulation in liver. Activities of ALT and AST were notably decreased by TSG treatment, and histological examination further presented the protective effect of TSG on livers in IR condition. Taken together, TSG protected liver from damage caused by abnormal lipid deposition, conducing to controlling IR.

In physiological state, insulin binding to insulin receptor leads to IRS-1 phosphorylation. It initiates insulin signal transduction and then promotes GLUT4 and glucose transport. During IR occurrence, the signaling of IRS-1/GLUT4 is inhibited. It has been well proved that AMPK serves as an important regulator in glucose and lipid metabolism [5, 46]. AMPK has been recommended as a target in the treatment of numerous diseases [7, 47]. Activation of AMPK has been shown to improve insulin sensitivity, attenuate gluconeogenesis, and ameliorate abnormal lipid metabolism [47–49]. Moreover, there were evidences showing that the expression of GLUT4 could be positively regulated by activated AMPK [8, 9]. As our results showed (Figure 5), the signaling pathways of IRS-1/GLUT4 and AMPK were suppressed in IR, bringing out disordered metabolism of glucose and lipid. TSG was found to repair the glucose metabolism and insulin signaling by promoting expression of IRS-1 and GLUT4. In the meantime, TSG noticeably reversed the suppression of AMPK activation, which attenuated gluconeogenesis and profited hepatic lipid metabolism and further prevented blood glucose from increasing and hepatic steatosis.

In the past decade, studies on insulin resistance have gradually revealed a relation between low-grade systematic inflammation and pathological insulin sensitivity, which was evidenced by changes in biochemical markers of inflammation [50, 51]. We observed the increased levels of TNF-*α*, IL-1*β*, and IL-6 in mice under IR condition, supporting the correlation between systemic inflammatory state and IR. These results were consistent with previous studies [52, 53]. TSG greatly reduced inflammatory cytokines, indicating that inflammation induced by HFD was alleviated due to TSG treatment.

Of wide knowledge, TRPV1 is a wide-expression calcium channel. TRPV1 can be activated by physical and chemical stimulation, mediating harmful reactions under pathological conditions [54]. Raised expression of TRPV1 on mRNA and protein levels has been reported in various inflammatory models [55, 56]. There was evidence providing that the level of TRPV1 expression was increased with exposure to TNF-*α* [57, 58]. TRPV1 can be stimulated by reactive oxygen species (ROS) as well and mediates generation of ROS and inflammatory mediators, contributing to inflammation and tissue damage [18, 59–61]. Furthermore, a range of studies demonstrated that application of TRPV1 deletion or TRPV1 antagonists could alleviate metabolism-related low-grade inflammation *in vivo*. HFD raised the levels of IL-1*β* and IL-6 in wild-type mice but had not affects in TRPV1-null mice, and lack of TRPV1 improved glucose tolerance though did no significant differ on weight gain [22]. Similarly, application of TRPV1 antagonists made obvious decrease in levels of serum proinflammatory markers (TNF-*α*, IL-6, and NF-*κ*B) and alleviated IR as well [62]. Our results supported a harmful function of increased TRPV1 expression in IR mice. Based on above findings, we suggested that TSG decreased the levels of proinflammatory cytokines, relieving chronic inflammation in IR mice, via suppressing TRPV1 expression.

On the other hand, oxidative stress has been proven to be another key even in the onset and progress of IR [63, 64]. Excessive fat intake makes lipid accumulation and peroxidation in body, producing superfluous ROS [65, 66]. And when the antioxidant system cannot overcome the generation of ROS, oxidative stress eventually occurs [67]. In the present study, the generation of MDA was increased whereas the activity of SOD was lowered in IR mice, implying the imbalance between oxidation and antioxidant capacity. We confirmed that TSG reversed the abnormal accumulation of MDA in serum and compromised activity of SOD, thus exhibited antioxidant effect.

SIRT6, a kind of deacetylases, has been reported to regulate a myriad of biologic processes [68–70]. SIRT6 inhibition has been established in clinical and animal models of IR [29, 71]. Mounting studies have believed that SIRT6 protected cells in many diseases including NAFLD, T2DM, and obesity based on IR [25, 72–74]. Overexpression of SIRT6 has been proven to reduce blood glucose levels in mice with both standard chow and high-calorie diet and enhance insulin sensitivity in mouse liver [75]. In recent years, SIRT6 has been identified as a coactivator of Nrf2, promoting activation of Nrf2 and its downstream enzymes against oxidative stress [23, 76]. It was found that diet-induced hepatic IR exacerbated in hepatocyte-specific SIRT6-knockout mice due to downregulation of Nrf2 as well as upregulation of Nrf2 functional repressor [29]. In the present study, we observed that TSG greatly upregulated hepatic protein expression of SIRT6, Nrf2, SOD1, and SOD2 in IR mice. Our results indicated that TSG from loquat leaves attenuated hepatic oxidative stress in IR mice by potentiating SIRT6/Nrf2 signaling and its downstream enzymes. Furthermore, it is worth noting that SIRT6 has been suggested to take a part in TRPV1-mediated inflammation. In the inflammatory state, the level of SIRT6 was decreased while TRPV1 expression was upregulated [30, 77]. Overexpression of SIRT6 could suppress TRPV1 and result in significant downregulation ROS and NF-*κ*B production, achieving anti-inflammatory effect [31]. It proved a support for the negative regulatory role of SIRT6 in TRPV1-mediated inflammation. Our results showed the low level of SIRT6 and high level of TRPV1 in IR that was accompanied with inflammation while TSG reversed the trend. From this point of view, further studies are required to explore the deep mechanism of TSG on anti-inflammation by TRPV1 and SIRT6.

## 5. Conclusion

Our study demonstrated that TSG from loquat leaves prevented HFD-induced IR by reducing abnormal lipid accumulation, inflammation, and oxidative stress. These effects were attributed to enhanced signaling of IRS-1/GLUT4 and activated AMPK as well as the modulation of TRPV1 and SIRT6/Nrf2 signaling pathways. Given the potential of IR amelioration by TSG in this study, TSG can be used to treat T2DM. And further studies can be performed with every monomer in TSG to evaluate the potential of each individual sesquiterpene glycosides on IR reduction *in vivo*.

## Data Availability

The data used to support the findings of this study are available from the corresponding author upon request.
